# Normal Weight Obesity and Cardiometabolic Risk Factors: A Systematic Review and Meta-Analysis

**DOI:** 10.3389/fendo.2022.857930

**Published:** 2022-03-24

**Authors:** Nami Mohammadian Khonsari, Patricia Khashayar, Ehsan Shahrestanaki, Roya Kelishadi, Sahar Mohammadpoor Nami, Motahar Heidari-Beni, Zahra Esmaeili Abdar, Ozra Tabatabaei-Malazy, Mostafa Qorbani

**Affiliations:** ^1^ Non-Communicable Diseases Research Center, Alborz University of Medical Sciences, Karaj, Iran; ^2^ Center for Microsystems Technology, Imec & Ghent University, Zwijnaarde-Gent, Belgium; ^3^ Social Determinants of Health Research Center, Alborz University of Medical Sciences, Karaj, Iran; ^4^ Department of Pediatrics, Child Growth and Development Research Center, Research Institute for Primordial Prevention of Non-Communicable Disease, Isfahan University of Medical Sciences, Isfahan, Iran; ^5^ Department of Nutrition, Child Growth and Development Research Center, Research Institute for Primordial Prevention of Non-Communicable Disease, Isfahan University of Medical Sciences, Isfahan, Iran; ^6^ Non-Communicable Diseases Research Center, Endocrinology and Metabolism Population Sciences Institute, Tehran University of Medical Sciences, Tehran, Iran

**Keywords:** normal weight obesity, central obesity, obesity, cardiometabolic, metabolic syndrome

## Abstract

**Background:**

Obesity is one of the most significant causes of morbidity and mortality worldwide. Current studies suggest a new type of obesity, normal weight obesity (NWO), which is defined as having a normal body mass index (BMI), but a high-fat percentage increases the risk of cardiometabolic risk factors (CMRFs). This systematic review and meta-analysis aimed to pool the association between NWO with CMRFs.

**Methods:**

A systematic search of the literature in all available electronic databases, including Scopus, Web of Science, EMBASE, and PubMed, was performed until October 2021. All English studies that assessed the association of NWOs [compared to normal weight non-obese (NWNO)] and the CMRFs were included. Two investigators extracted data and performed a quality assessment. The heterogeneity between studies was assessed with I-squared and Cochran’s Q tests. Odds ratio (OR) was used as an effect size to pool the association of NWO with CMRFs.

**Results:**

Twenty-five articles that met the inclusion criteria entered the study. The total number of participants was 177,792, with an age range of 13 to 75 years. Most studies were conducted on the general population (adults) and were from China. The result of fixed-effect model meta-analysis indicated an increased odds of hyperglycemia (OR:1.50, 95%:1.23, 1.76), high TG (OR:1.90, 95% CH:1.44, 2.35), low HDL (OR: 1.28, 95% CI:1.06, 1.49) and diabetes (OR:1.39, 95% CI:1.30, 1.49). Moreover, the random effect meta-analysis showed that NWO increased the odds of dyslipidemia (OR:1.83, 95% CI:1.61, 20.4), HTN (OR:1.40, 95% CI:1.28, 1.51) and metabolic syndrome (OR:1.92, 95% CI:1.58, 2.26). Moreover, the mean of all CMRFs except plasma glucose in NWO subjects was statistically higher than NWNO subjects (p-value<0.05).

**Conclusion:**

The present study showed that NWO increased the odds of CMRFs. These findings indicate the inadequacy of the BMI measurement and the need for body fat assessment for a better obesity risk assessment.

## Background

Obesity is one of the most significant causes of morbidity and mortality worldwide (1, 2). In literature, obesity is usually defined as a body mass index (BMI) above 30 Kg/m^2^ (2). The prevalence of obesity is increasing throughout the globe. This disease imposes a significant burden on the affected population and the health system. It is also considered a fulcrum of other conditions, such as cardiometabolic conditions, that arise from obesity and are the leading cause of death worldwide (1–3). Although the prevalence of these supposedly obesity-related complications (e.g., diabetes, hypertension, dyslipidemia, etc.) and cardiometabolic diseases is exceptionally higher among obese individuals, their prevalence has been increased in the past few decades, among the non-obese population (BMI under 30 Kg/m^2^) and even in those considered healthy based on their BMI levels (BMI between 18.5 to 24.9 Kg/m^2^) (4–6). This shows that BMI, long known as a great assessment tool, cannot determine an individual’s body composition, and fat percentage, lacking the adequate properties to identify those with a high body fat percentage or disproportionate body fat distribution (e.g. abdominal obesity) (7) Recent studies suggest the percentage of body fat is directly related to cardiometabolic and obesity-related conditions. This is a new type of obesity in which an individual with normal BMI levels is considered as obese based on their body composition and fat percentage (8, 9). Normal weight obesity (NWO) has different definitions based on the studies, population, and gender; however, it is usually defined as a body fat percentage above 30% (10). Due to the lifestyle changes, lack of proper physical activity and the use of processed food, the numbers of obese individuals are on the rise (11, 12); accordingly, the number of the normal weigh obese might be increasing; however, due to their normal BMI they will remain undiagnosed, and no proper preventive measure is taken until it is too late (13). Since there has been no new individual data or aggregated systematic reviews and meta-analyses on this relatively novel subject, we conducted this study to assess the cardiometabolic risk factors (CMRFs) and anthropometric measurements in the NWO individuals and compare them with the normal population. This study aims to give a realistic overview of the emerging obesity-related conditions so that health authorities can take proper action and implement appropriate preventive measures.

## Methods

This study was conducted according to the Preferred Reporting Items for Systematic Review and Meta-Analysis (PRISMA) guidelines.

### Search Strategy

We conducted a systematic search of the literature in all available electronic databases, including Scopus, Web of Science, EMBASE, and PubMed, until October 2021. The terms used for the search was “NWO”, “central obesity”,” high-fat percentage”, and their equivalent terms based on MesH terms. The search strategy is presented in the **Supplementary Table 1**. Moreover, one investigator conducted the search, and another investigator reviewed the search results.

### Eligibility Criteria and Selection Study

All English studies that assessed anthropometric measurements and the CMRFs [BMI, lean body mass, body fat mass, waist, hip, plasma glucose level, total cholesterol, Homeostatic Model Assessment for Insulin Resistance (HOMA), low-density lipoprotein (LDL), High-density lipoprotein (HDL), Triglyceride (TG), Total cholesterol (TC), systolic blood pressure (SBP), diastolic blood pressure (DBP), hypertension (HTN)] among NWO individuals were included. All studies had to represent the target population and compare them with the normal-weight non-obese (NWNOs) individuals and adjust for possible confounders to be included in our study. Only the most recent studies were included in our research if multiple studies used the same data source. All definitions of NWO, Regardless of their variety (e.g. normal BMI with high body fat percentage, normal BMI with high waist circumference (central obesity), high waist to hip/height ratio and etc.) were included in this study.

After removing the duplicates using EndNote X7, two investigators independently assessed the titles, abstracts, and finally, the full texts of the remaining articles. In addition, hand searching was performed to find relevant studies from the reference list of the included articles. Any discrepancies were referred to the third investigator for resolution.

### Data Extraction Strategy

Two investigators independently extracted the data using an electronic data extraction sheet. The extracted data included the name of the first author, the year of the study, sample size, sex, age (mean or range), NWO definition, CMRFs, Odds ratios (OR) or standard mean difference (SMD), and 95% confidence interval (CI) as an effect size of dichotomous and continuous data respectively. Two other investigators helped resolve any discrepancy.

### Quality Assessment (QA)

The Newcastle-Ottawa Scale was used for the quality assessment of the included articles. This seven-item scale scores the selection, exposure (case-control study) and outcome (cohort study), and comparability of the studies. The total score, which is the sum of each item score, ranges from 0 to 9, with greater scores indicating lower bias risk. The scores were categorized as 0 to 4, 5 and 6, 7 to 9, meaning unsatisfactory, satisfactory, and good quality, respectively. All of the above steps were assessed independently by two investigators. Finally, any discrepancies were referred to the third investigator for resolution.

### Statistical Analysis

The heterogeneity between the studies was assessed using the I-squared and Cochran’s Q tests if heterogeneity was statistically significant (P-value<0.1) (14). a random effect model was used; otherwise, a fixed model was applied. Odds Ratio (OR) and 95% confidence interval (CI) were used as an effect size of meta-analysis to pool the association of NWO with CMRFs as a dichotomous variable. We also calculated and pooled the standardized mean difference (SMD) as an effect size for NWO association with the means of CMRFs. Meta-analysis was done for outcomes that were reported in more than three studies. Sub-group analysis was performed for the CMRFs. Publication bias was assessed using Egger’s test for each CMRF; if publication bias was seen, sensitivity analysis was performed. STATA version 11 (Stata Corporation, College Station, Texas, USA) was used for the analysis.

## Results

### Search Results

**Figure 1:**
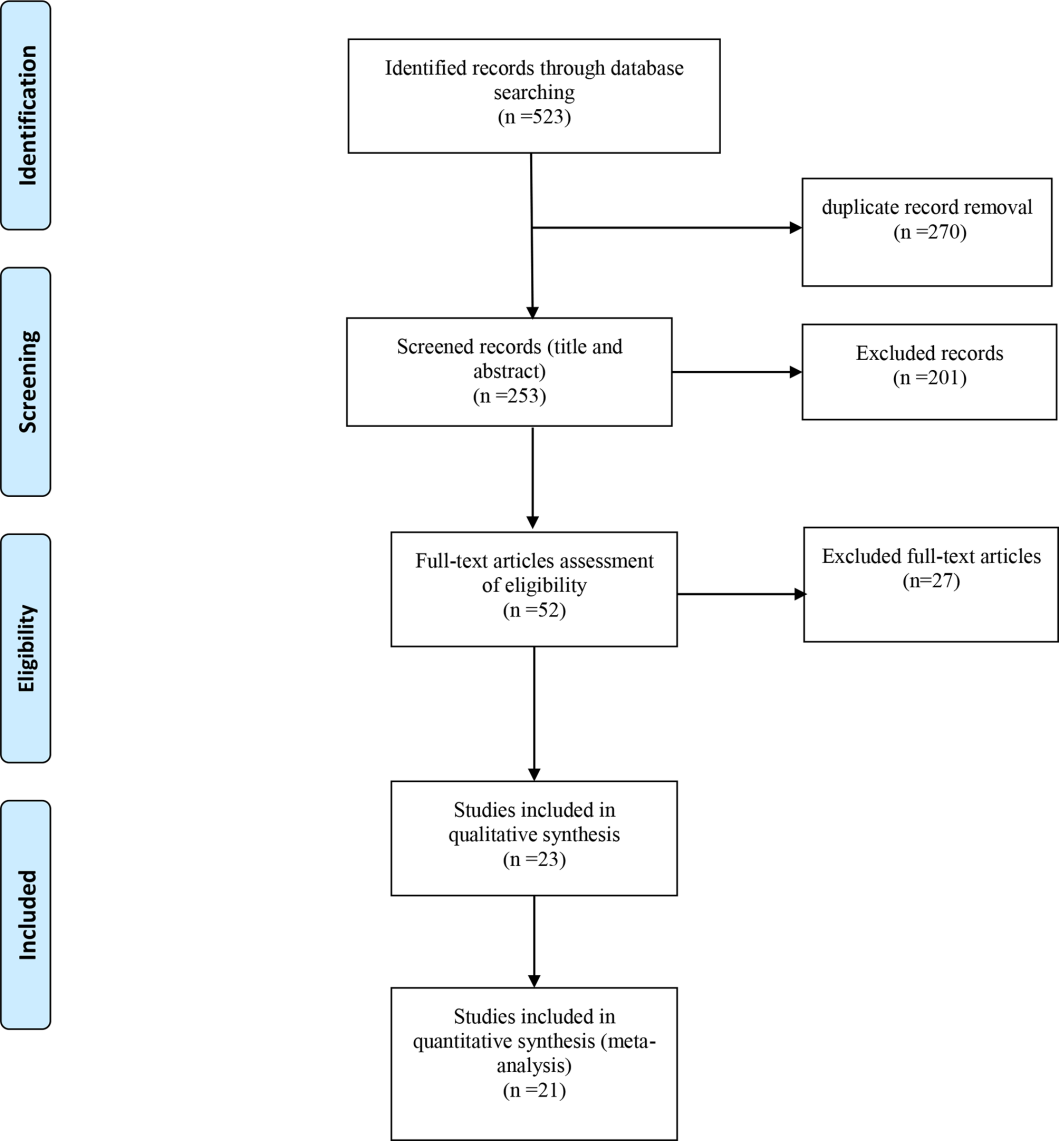
Studies search and review flowchart.

From the 523 studies of the initial search, 270 were duplicates; thus, 253 articles were evaluated, and 201 were considered irrelevant based on the title and abstract. The remaining 52 articles’ full text was then assessed and evaluated for eligibility criteria, and 27 articles were excluded. Twenty-three articles met the inclusion criteria. Two studies with unadjusted data for potential confounders were included due to their exceptionally high, quality assessment score; however, these two studies were not included in the Quantitative synthesis. This process is illustrated in **Figure 1**.

### General Characteristics

These studies were conducted worldwide (United States of America, Sweden, Korea, Colombia, West Indies, China, India, Iran, Japan, Iceland, Malaysia, Switzerland, Brazil, and Finland). The total number of participants was 177,792, with an age range of 13 to 75 years. These data, alongside other study characteristics, are presented in **Table 1**. Most of these studies were conducted on the general population (adults) and were from china (5 studies) and Korea (4 studies). With one study, Sweden, West Indies, India, Iran, Japan, Iceland, Malaysia, Switzerland, and Finland had the lowest number of studies. The largest sample size was from Japan with 117163 participants, and the smallest was from Iceland with 182 participants (4, 6, 8, 15–36).. These general characteristics of included studies for NWO association with CMRFs are shown in **Table 1**.

**Table 1:** General Characteristics of included studies for association of NWO with CMRFs.

Author Year	Country	sample size					Mean Age/Age Range	Study Population	Definition of NWO*	Q.A
		Total	M	F	NWNO	NWO				
Bellissimo 2019 (15)	USA	289	63	116	26	43	47	Adults/general population	BF: 23% men, 30% women	6
Berg 2015 (16)	Sweden	1471	581	890	1080	266	25-74	Adults /general population	BF: 25% men , 38% women	8
W. K. Cho 2015 (17)	Korea	1700	888	812	1266	144	13-18	Adolescents	upper highest quartile (Q4) of age and sex specific Waist-to-height ratio	8
M. Correa 2020 (18)	Colombia	1354	528	826	961	393	18-32	Adults /general population	BF: 25.5% men , 38.9% women	9
Ramsaran 2017 (19)	West Indies	236	76	160	189	74	18-32	Adults /general population	BF: 23% men , 33% women	6
A. García 2020 (20)	Colombia	1919	955	964	1035	884	13	Children and Adolescents	BF: boys > 23.4%–28.3% and girls > 31.0%–34.1%	7
H. He 2019 (21)	China	2654	(–)	(-)	1916	729	46.9 ± 1380	Adults /general population	BF: 21.4% men , 31.4% women	9
A. Jia 2018 (22)	China	15291	1492	13799	9988	1771	under 75	Adults / general population	BF: ≥24% for men and ≥33% for women	10
Kapoor 2020 (23)	India	1147	619	528	200	364	47.3 ± 7.5	Adults /general population	BF: , ≥20.6% men, ≥33.4% women,	9
Kim 2014 (24)	Korea	12217	5313	6904	3382	1575	44	Adults /general population	BF: ≥20.6% men, ≥33.4% women	8
Kim 2018 (25)	Korea	3949	(–)	(-)	2213	868	(-)	Adults /general population	BF: ≥23.1% men, ≥33.1% women	6
Sohee Kim 2015 (26)	Korea	2078	1141	937	1795	283	53.4	Adults /general population	BF: ≥25.4 % men, ≥31.4 % women	8
H. Zhao 2012 (27)	China	407	(-)	(-)	(-)	(-)	(-)	(-)	BF: ≥25 % men, ≥35 % women	5
Tayefi 2019 (28)	Iran	2439	(–)	(-)	1311	1128	47	Adults /general population	BF: > 25%men, >30% women	9
T. Shirasawa 2019 (8)	Japan	117163	82487	34676	43055	12877	40-64	Adults /general population	Waist-to-height ratio ≥ 0.5	10
A. Romero 2010 (6)	USA	4116	2089	2027	2054	2062	41.3	Adults /general population	BF: ≥23.1 % men, ≥33.3 % women	8
A. S. Olafsdottir 2016 (29)	Iceland	182	96	86	106	76	17.7-18.9	High-school students/ adolescents	BF: > 17.6% men , >31.6% women	5
F. M. Moy 2015 (30)	Malaysia	858	0	858	511	237	40.47 ± 8.9	Adults /teachers	BF: >28.52%	6
K. E. Martinez 2017 (31)	USA	3600	(-)	(-)	1624	288	adults	Adults /general population	BF: 27.8 ± 0.2% men, 40.5 ± 0.2 % women	5
P. Marques-Vidal 2010 (32)	Switzerland	2301	0	2301	1667	173	35-75	Adults/general population whites	BF: ≥38%	7
F. B. Madeira 2013 2013 (4)	Brazil	1222	546	676	1111	111	23-25	young adults	BF: ≥23% men, ≥30% women	10
P. J. Liu 2017 (33)	China	412	0	412	214	198	55.72	Post menopause general population women	third tertile of normal weight body fat	6
C. C. N. Da Silva 2021 (34)	Brazil	787	346	441	553	47	23-25	Young adults	>90th percentile body fat	10

CMRFs, cardio-metabolic risk factors; M,male; F, female; NWNO, normal weight none obese; NWO, normal weight obese; BF, body fat (based on percentage); USA, United State; Q.A, quality assessment based on the Newcastle Ottawa scale (out of 10 points); * all NWOs had BMIs within normal range.

### Qualitative Synthesis

The association of NWO with the means of CMRFs compared to NWNO in included studies is shown in **Table 2** alongside their effect size. As illustrated, most anthropometric components among NWOs significantly differed from that of NWNOs with the most significant, regarding the fat mass [effect size: -1.9 95%.CI (-2.01_-1.8)]. The reported mean range of the associated cardiometabolic parameters are as follows, plasma glucose 81.96 to 95.7 mg/dL for NWNOs and 80.47 to 99.1 mg/dL for NWOs. Total cholesterol, 181.61 to 204.97 mg/dL for NWNOs and 189.61 to 216.55 mg/dL for NWOs, HOMA, 0.6 to 3.32 for NWNOs and 0.8 to 3.18 for NWOs, LDL, 105.3 to 121.8 mg/dL for NWNOs and 117.67 to 132.64 mg/dL for NWOs, HDL mg/dL, 42.09 to 72.7 mg/dL for NWNOs and 45.72 to 69.5 mg/dL for NWOs, TG, 76.72 to 116.03 mg/dL for NWNOs and 85.32 to 136.4 mg/dL for NWOs, SBP, 107.81 to 129 mmHg for NWNOs and 110.58 to 134 mmHg for NWOs, DBP, 71 to 80 mmHg for NWNOs and 72.1 to 85 mmHg for NWOs. As for anthropometrics, the mean ranges of lean mass were 40.21 to 57.5 kg in NWNOs and 39.9 to 57.4 kg in NWOs, waist, 59.12 to 84.4 cm for NWNOs and 63.94 to 89.9 in NWOs, hip 93.2 to 96.1 cm in NWNOs and 94.6 to 98.9 cm NWOs. Association of NWO with CMRFs as categorical data in included studies are shown in **Table 3**. As illustrated, most reported ORs are statistically significant. The greatest reported OR was of elevated waist circumference (WC) among NWOs [OR: 26.61 95%.CI (4.75-149.14)] and the odds of metabolic syndrome (MetS) among NWOs [OR:8.89 95%.CI (3.32-4.47)].

**Table 2:** Association of NWO with mean of CMRFs in included studies.

Author, year	Outcome	NWNO		NWO		Effect size		Adjustment
		Mean	SD	Mean	SD	SMD	95% CI	
Bellissimo, 2019 (15)	Plasma glucose	95.7	24	93.1	26.2	0.1	-0.39_0.59	Sex, age and ethnicity
	Plasma insulin	2.7	2.5	3.6	3.2	-0.3	-0.79_0.19	
	TC	193.9	47	201.4	52.4	-0.15	-0.63_0.34	
	HOMA	0.6	0.5	0.8	0.65	-0.33	-0.82_0.16	
	LDL	105.3	41	117.8	45.85	-0.28	-0.77_0.21	
	HDL	72.7	19	63.5	21.61	0.44	-0.05_0.93	
	TG	81.1	57	101.2	62.22	-0.33	-0.82_0.16	
	SBP	119.4	18	118.1	20.3	0.07	-0.42_0.55	
	DBP	74.7	12.5	75.5	13.75	-0.06	-0.55_0.43	
	BMI	23.9	4.59	24.3	4.55	-0.09	-0.57_0.40	
	Lean mass*	50.3	7.65	44.4	16.25	0.43	-0.07_0.92	
	Fat mass*	16.5	9.18	21.5	9.75	-0.52	-1.01_-0.02	
berg, 2015 (16) (male)	Plasma glucose	91.8	17.83	93.6	10.82	-0.11	-0.31_0.08	Age
	TC*	201.08	38.33	216.55	46.52	-0.38	-0.58_-0.18	
	LDL*	119.69	33.28	131.27	50.07	-0.3	-0.5_-0.1	
	HDL*	61.78	0	57.92	23.24	0.32	0.12_0.52	
	TG*	88.57	307.09	106.28	53.29	-0.07	-0.26_0.13	
	SBP*	129	19.81	134	18.04	-0.26	-0.45_-0.06	
	DBP*	80	9.9	85	9.02	-0.52	-0.71_-0.32	
	BMI*	22.8	2.47	24.1	2.7	-0.51	-0.71_-0.32	
	Lean mass	57.5	5.94	57.4	5.41	0.02	-0.18_0.21	
	Fat mass*	15	2.97	20.6	4.51	-1.62	-1.84_-1.4	
	Waist*	84.4	7.92	89.8	7.51	-0.69	-0.89_-0.49	
	Hip*	96.1	5.44	98.9	5.71	-0.51	-0.7_-0.31	
Berg, 2015 (16) (female)	Plasma glucose	88.2	12.17	88.2	10.34	0	-0.19_0.19	
	TC*	204.97	52.28	216.55	33.34	-0.23	-0.42_-0.04	
	LDL*	111.97	52.21	119.69	33.29	-0.16	-0.34_0.03	
	HDL	69.5	26.1	69.5	22.19	0	-0.19_0.19	
	TG*	88.57	59.92	97.42	50.91	-0.15	-0.34_0.04	
	SBP	125	13.52	125	17.24	0	-0.19_0.19	
	DBP	80	13.52	80	8.62	0	-0.19_0.19	
	BMI*	22.1	2.7	24.1	2.87	-0.73	-0.93_-0.54	
	Lean mass	42.8	4.05	42.9	4.02	-0.02	-0.21_0.16	
	Fat mass*	17.6	4.05	23.3	4.88	-1.36	-1.56_-1.16	
	WC*	60.6	8.11	66	7.76	-0.67	-0.86_-0.48	
	Hip*	94.8	7.44	98.9	6.61	-0.56	-0.75_-0.37	
A. García, 2020 (20) (male)	Plasma glucose	83.36	14.95	81.91	17.04	0.09	-0.04_0.22	Age, BMI and pubertal stage
	HDL	49.6	12.99	45.72	11.75	0.31	0.18_0.44	
	TG*	76.72	30.8	85.32	38.13	-0.25	-0.38_-0.12	
	SBP	111.44	14.98	111.46	13.34	0	-0.13_0.13	
	WC*	61.78	5.12	65.85	6.13	-0.73	-0.86_-0.59	
A. García, 2020 (20) (female)	Plasma glucose	81.96	15.16	80.47	16.86	0.09	-0.03_0.22	
	HDL	49.63	12.51	47.15	12.21	0.2	0.08_0.32	
	TG*	87.16	36.61	97.86	62.59	-0.21	-0.34_-0.09	
	SBP	107.81	12.31	110.58	12.36	-0.22	-0.35_-0.1	
	WC*	59.12	5.07	63.94	5.89	-0.88	-1.01_-0.75	
A. García, 2020 (20)	BMI*	17.86	1.85	19.67	1.91	-0.96	-1.06_-0.87	
	Fat mass*	7.24	3.09	9.89	3.56	-0.8	-0.89_-0.71	
Sohee kim, 2015 (26)	Plasma glucose *	95.7	17.5	99.1	15.9	-0.2	-0.32_-0.07	Age, sex, and smoking status
	TC*	192.5	34.6	199.6	35.5	-0.2	-0.33_-0.08	
	LDL*	117.1	30.6	123.9	32.2	-0.22	-0.35_-0.1	
	HDL*	51	12.8	49.6	11.3	0.11	-0.01_0.24	
	TG*	106.4	65.1	116.6	63.3	-0.16	-0.28_-0.03	
	SBP*	122.5	15.5	128.6	15.7	-0.39	-0.52_-0.27	
	DBP*	76.6	10.1	79.6	9.3	-0.3	-0.43_-0.17	
	BMI*	22.53	4.3	23.9	0.8	-0.34	-0.47_-0.22	
Tayefi, 2019 (28)	Plasma glucose	86.38	35.66	91.3	40.4	-0.13	-0.21_-0.05	Age and sex
	TC	181.93	38.16	189.61	39.14	-0.2	-0.28_-0.12	
	LDL	113.19	33.54	117.67	35.06	-0.13	-0.21_-0.05	
	HDL*	42.09	9.8	45.74	9.43	-0.38	-0.46_-0.3	
	TG*	105	738.93	126	753.96	-0.03	-0.11_0.05	
	SBP*	116.26	16.39	118.1	19.9	-0.1	-0.18_-0.02	
	DBP*	76.35	11.84	75.75	11.85	0.05	-0.03_0.13	
	BMI*	22.62	3.38	23.39	4.18	-0.2	-0.28_-0.12	
	WC*	84.52	9.89	86.01	11.53	-0.14	-0.22_-0.06	
	Hip*	94.22	6.91	96.88	8.86	-0.34	-0.42_-0.26	
A. Romero, 2010 (6) (male)	Plasma glucose	95.6	15.92	96.8	22.32	-0.06	-0.15_0.03	Age and race
	HOMA*	0.84	0.31	1	0.63	-0.32	-0.41_-0.23	
	LDL*	121.8	49.03	132.64	49.11	-0.22	-0.31_-0.13	
	HDL*	49.11	12.1	47.56	12.11	0.13	0.04_0.22	
	TG*	116.03	84.38	113.75	84.51	0.03	-0.06_0.11	
	SBP	122	15.92	125	15.94	-0.19	-0.28_-0.1	
	DBP	74	12.73	76	9.57	-0.18	-0.26_-0.09	
	BMI*	22.7	1.27	23.5	1.27	-0.63	-0.72_-0.54	
	WC*	84.8	6.05	88.9	6.37	-0.66	-0.75_-0.57	
	Lean mass*	55.4	5.73	53	5.74	0.42	0.33_0.51	
	Fat mass*	14.6	1.59	18.5	2.55	-1.83	-1.94_-1.73	
	Hip*	93.2	4.13	94.6	4.14	-0.34	-0.43_-0.25	
A. Romero, W2010 (6) (female)	Plasma glucose	92	22.57	91.1	22.62	0.04	-0.05_0.13	
	HOMA*	0.87	0.32	0.98	0.64	-0.22	-0.3_-0.13	
	LDL*	116.4	49.66	124.13	62.39	-0.14	-0.22_-0.05	
	HDL	58	12.25	57.62	12.28	0.03	-0.05_0.12	
	TG*	100.97	85.46	136.4	228.87	-0.2	-0.29_-0.12	
	SBP	117	19.34	119	20.04	-0.1	-0.19_-0.02	
	DBP	71	9.67	72.1	10.34	-0.11	-0.2_-0.02	
	BMI*	22.1	1.29	23.5	0.97	-1.23	-1.32_-1.13	
	WC*	78.3	6.45	83.3	6.46	-0.77	-0.86_-0.69	
	Lean mass*	40.21	4.19	39.9	3.55	0.08	-0.01_0.17	
	Fat mass*	18.1	1.93	22.1	2.26	-1.9	-2.01_-1.8	
	Hip*	94.4	4.51	97.7	4.85	-0.7	-0.79_-0.62	
K. E. Martinez, 2017 (31)	HOMA*	1.1	2.01	1.6	1.52	-0.26	-0.38_-0.13	Age, sex, race, and year of assessment. moderate physical activity, vigorous physical activity, and smoking
P. Marques-Vidal, 2010 (32)	Plasma insulin	8.56	13.88	8.17	6.97	0.03	-0.13_0.19	Age
	HOMA	3.32	5.71	3.18	2.89	0.03	-0.13_0.18	
	BMI*	21.8	4.08	23	2.63	-0.3	-0.46_-0.15	
	WC*	75.9	8.16	79.9	7.89	-0.49	-0.65_-0.33	
	Hip*	94.4	8.16	96.1	6.57	-0.21	-0.37_-0.06	

CMRFs, cardio-metabolic risk factors; NWNO, normal weight non obese; NWO, Normal weight obesity; M, male; F, female; TC, total cholesterol; HOMA, Homeostatic Model Assessment for Insulin Resistance; LDL, low-density lipoproteins; HDL, high-density lipoproteins; TG, triglyceride; SBP, systolic blood pressure; DBP, diastolic blood pressure; BMI, body mass index; WC, waist circumference; SMD, Standardized Mean Difference; SD, Standard Deviation; CI, Confidence Interval. Plasma glucose, TC, LDL, HDL, TG values are reported in the mg/dL unit. SBP and DBP in mmHg, BMI In kilograms by height (in meters) squared, lean and fat masses in kilograms, Hip and WC in centimeters.

*Statistically significant (p-value < 0.05).

The SMDs were calculated based on Hedges’ g formula.

**Table 3:** Association of NWO with CMRFs in included studies.

Author Year	Outcome	Definition of outcome	OR (95% CI)**	Adjustment
W. K. Cho 2015 (17) (Male)	HOMA	Fasting glucose (in millimoles per liter) × fasting insulin (in milliunits per liter)/22.5	2.46 (1.21-4.99)	Age, weight, and ALT
W. K. Cho 2015 (17) (Female)	HOMA		1.51 (0.83-2.75)	
M. Correa 2020 (18)	hyperglycemia	FBS ≥5.6 mmol/L [100 mg/dL]	1.31 (0.73-2.33)	Age and sex
	HTN	≥130 mm Hg SBP and/ or DBP 85 mm Hg	1.42 (0.89-2.27)	
	Elevated TG	≥1.7 mmol/L [151 mg/dL]	1.31 (0.62-2.76)	
	Elevated LDL	≥2.6 mmol/L [100 mg/dL]	1.27 (0.85-1.90)	
	Reduced HDL	low HDL: < 1 mmol/L [38.7 mg/dL] in men and 1.3 mmol/L [50.3 mg/dL] in women	2.34 (1.61-3.93)*	
	Cardiometabolic risk Z-score	+ 1 SD above the mean	3.10 (2.06-4.67)*	
	obesity	Waist to hip ratio > 0.49 in men and > 0.50 in women	2.61 (0.69-9.87)	
	Abdominal Obesity	WC ≥ 90 cm in men, and ≥ 80 cm in women	7.27 (1.09-48.60)*	
Ramsaran 2017 (19)	Elevate DBP	high SBP and DBP systolic ≥ 120 mm Hg and the diastolic ≥ 80 mm Hg	0.98 (0.39-2.48)	Not adjusted
	Elevate SBP		1.85 (0.52-5.52)	
	Elevated WC	men ≥ 94 cm and women ≥ 80 cm	26.61 (4.75-149.14)*	
H. He 2019 (21)	HTN	SBP ≥ 140 and,or DBP ≥ 90	1.82 (1.43-2.30)*	Age, sex, social economic profiles, lifestyle factors, family history of HTN and other disease status, etc.
A. Jia 2018 (22)	DM	FBS 7.0 ≥ mmol/L; blood glucose 2 h after an OGTT ≥ 11.1 mmol/L; a previous diagnosis of diabetes; or current use of hypoglycemic agents	1.44 (1.10–1.88)*	Age , sex, ethnicity, smoking, alcohol use, exercise, education, yearly family income, family history of disease, and WC
	HTN	SBP ≥ 130 and,or DBP ≥85	1.53 (1.27–1.84)*	
	MetS	IDF^1^	1.48 (1.22–1.79)*	
	Elevated Framingham risk	Score ≥ 10%	2.36 (1.76–3.17)*	
Kapoor 2020 (23)	DM	FBS ≥ 126 mg/dl and/or 2-h plasma glucose value of ≥ 200 mg/dl were diagnosed to have diabetes/	2.72 (1.46–5.08)*	Age, sex, tobacco use and alcohol intake
	HTN	SBP ≥ 140 and, or DBP ≥ 90	1.89 (0.92–3.86)	
	Dyslipidemia	taking lipid-lowering medications and/or TC >200 mg/dl and/or LDL >100 mg/dl and/or HDL <40 mg/dl in men and <50 mg/dl in women and/ or TG >200 mg/dl.	2.37 (1.55–3.64)*	
Kim, 2014 (24) (Male)	DM	fasting blood glucose ≥ 126 mg/dl or treatment of the disease	1.38 (1.04 -1.83)*	Age and lifestyle factors
	HTN	SBP ≥ 140 and, or DBP ≥ 90	1.70 (1.42 - 2.02)*	
	dyslipidemia	total cholesterol ≥ 240 mg/dl and/or high-density lipoprotein (HDL) cholesterol <40 mg/dl and/or triglyceride ≥ 150 mg/dl or treatment of dyslipidemia**	2.69 (2.29 - 3.17)*	
	MetS		2.50 ( 2.10 - 2.97)*	
	Mets risk factor above 1	plus 1 metabolic risk odds	3.54 ( 2.89 - 4.34)*	
Kim, 2014 (24) (Female)	DM	fasting blood glucose ≥ 126 mg/dl or treatment of the disease	1.72 (1.30 - 2.29)*	
	HTN	SBP ≥ 130 mmHg and, or DBP ≥85 mmHg	1.52 (1.25 - 1.86)*	
	dyslipidemia	total cholesterol ≥ 240 mg/dl and/or high-density lipoprotein (HDL) cholesterol <40 mg/dl and/or triglyceride ≥ 150 mg/dl or treatment of dyslipidemia**	1.70 (1.40 - 2.06)*	
	MetS		1.80 (1.48 - 2.20)*	
	Mets risk factor above 1	plus 1 metabolik risk odds	2.47 (2.01 - 3.03)*	
Kim 2018 (25)	MetS	IDF	1.83 (1.21 - 2.76)*	Potential confounders
	Plaque formation risk	(–)	1.46 (1.027 - 2.07)*	
H. Zhao 2012 (27)	HTN risk	(-)	2.18*	Age and sex
	Hyperglycemia		2.12*	
	dyslipidemia		2.08*	
	Hyperuricemia		3.49*	
Tayefi 2019 (28)	Risk of metabolically abnormal phenotype	(-)	2.02 (1.68-2.42)*	Age and sex
	Cardiac risk (Q)	QRISK calculated online by using the Framingham risk equation	6 (4.45-8.08)*	
T. Shirasawa 2019 (8) (Male)	DM	FBS ≥ 126 mg/dl, random plasma glucose ≥ 200 mg/dl, HbA1c (National Glycohemoglobin Standardization Program) ≥ 6.5%, or receiving medical treatment for DM	1.35 (1.25-1.46)*	Age, weight, smoking status, alcohol intake, and physical activity
	HTN	SBP ≥ 140 and, or DBP ≥ 90 or taking medication for HTN	1.22 (1.17-1.27)*	
	Dyslipidemia	as LDL-C ≥ 140 mg/dl, HDL-C < 40 mg/dl, TG ≥ 150 mg/dl, or taking medication for dyslipidemia	1.84 (1.74-1.89)*	
T. Shirasawa 2019 (8) (Female)	DM	FBS ≥ 126 mg/dl, random plasma glucose ≥ 200 mg/dl, HbA1c ≥ 6.5%, or receiving medical treatment for DM	1.60 (1.35-1.90)*	
	HTN	SBP ≥ 140 and,or DBP ≥ 90 or taking medication for hypertension	1.23 (1.16-1.31)*	
	dyslipidemia	as LDL-C ≥ 140 mg/dl, HDL-C < 40 mg/dl, triglycerides ≥ 150 mg/dl, or taking medication for dyslipidemia	1.60 (1.52-1.69)*	
A. S. Olafsdottir (29)	Mets	IDF	2.2 (1.2-3.9)	not adjusted
F. M. Moy 2015 (30)	Elevated TG	TG ≥ 1.7 mmol/L	2.51 (1.47–4.29)*	age and ethnicity
	Reduced HDL	HDL-C ≤ 1.3 mmol/L in women	1.09 (0.75–1.58)	
	Hypertension	systolic ≥130 mmHg and/or diastolic ≥85 mmHg or on antihypertensive treatment	1.63 (1.15–2.31)*	
	Hyperglycemia	FBG ≥ 5.6 mmol/L	1.67 (0.90–3.08	
	Mets	IDF	1.70 (0.87–3.32)	
	Hypercholesterolemia	(–)	2.22 (0.21–23.20)	
	Diabetes	Hyperglycemia: FBG ≥ 5.6 mmol/L.	1.28 (0.34–4.92)	
P. Marques-Vidal 2010 (32)	Elevated TG	TG ≥ 1.7 mmol/L [151 mg/dL] and/or LDL ≥ 2.6 mmol/L [100 mg/dL] (in the presence of myocardial infarction, stroke, coronary artery disease or diabetes) and ≥ 4.2 mmol/L [163 mg/dL] in other cases and/or hypolipidaemic drug treatment	2.21 (1.43-3.42)*	
	Low HDL	1 mmol/L in men and 1.3 mmol/L in women	2.10 (1.23-3.57)*	
	HTN	(–)	1.38 (0.97-1.98)*	
	Hyperglycemia	fasting hyperglycemia; HOMA > 4.88 (90th percentile in men) or >3.57 (90th percentile in women)	1.63 (1.10-2.42)*	
	dyslipidemia	HDL < 1 mmol/L in men and 1.3 mmol/L in women and/or TG ≥ 1.7 mmol/L [151 mg/dL] and/or LDL ≥ 2.6 mmol/L [100 mg/dL] (in the presence of myocardial infarction, stroke, coronary artery disease or diabetes) and ≥ 4.2 mmol/L [163 mg/dL] in other cases and/or hypolipidaemic drug treatment	1.90 (1.34-2.68)*	
	CMRF	the presence of at least two of the following: HTN ; TG ≥ 1.7 mmol/L; HDL cholesterol < 1 mmol/L [38.7 mg/dL] in men and 1.3 mmol/L [50.3 mg/dL] in women; fasting hyperglycemia; HOMA > 4.88 (90th percentile in men) or >3.57 (90th percentile in women) and CRP > 5.2 mg/L (90th percentile in men) or >6.1 mg/L (90th percentile in women) , definition 1. A second definition of metabolic risk was also applied, using the same criteria but with HOMA > 5.0 and CRP > 4.0 mg/L	1.37 (0.97-1.95)*	
	Abdominal obesity	(–)	2.64 (1.73–4.04)*	
F. B. Madeira 2013 (4)	Elevated TG	TG ≥ 150 mg/dL, use of lipid medications or self-reported diagnosis of hypertriglyceridemia	1.89 (0.97-3.70)	age, sex, skin colour, early and adult life variables (alcohol consumption, family income, schooling, marital status, smoking, percentage of fat in the diet and physical activity
	Reduced HDL	HDL < 40 mg/dL for men and ,50 mg/dL for women	1.53 (1.00-2.34)	
	HTN	SBP ≥ 130 mmHg and/ DBP ≥ 85 mmHg, current usage of antihypertensive drugs or previous diagnosis of hypertension	1.17 (0.65-2.13)	
	Hyperglycemia	high fasting blood glucose (≥100 mg/dL), current use of anti-diabetic medication or previously diagnosed diabetes	2.68 (1.01-7.12)*	
	HOMA	(–)	4.91 (1.85-13.04)*	
	Mets	IDF	8.89 (3.32-4.47)*	
	Elevated WC	central obesity (WC ≥ 90 cm for men and ≥ 80 cm for women	9.27 (5.32-16.15)*	
P. J. Liu 2017 (33)	Elevated TG	TGs ≥1.7 mmol/L	2.13 (1.10-4.12)*	age, smoking status ,drinking status, total cholesterol, LDL-c, high sensitivity C-reactive protein, and the remaining non-adipose MetS components, body fat percentage
	Reduced HDL	HDL <1.30 mmol	1.04 (0.61-1.75)	
	HTN	blood pressure ≥130/85 mmHg or current antihypertensive medication use	2.06 (1.09-3.90)*	
	Hyperglycemia	FBS ≥5.6 mmol/L, type 2 diabetes mellitus previously diagnosed by a physician, or current antidiabetic medication use	1.44 (0.77-2.68)	
	Mets risk factor above 2	IDF	2.00 (1.19-3.33)*	
C. C. N. Da Silva 2021 (34)	Elevated TG	TG levels above 150 mg/dL or use of lipid-lowering drugs	1.77 (1.12-2.79)*	total calories, family income, added sugar intake, total lipids intake, and physical activity
	Reduced HDL	<40 mg/dL for men and <50 mg/dL for women or use of lipid-lowering drugs	1.27 (0.98-1.65)	
	HTN	SBP > 130 mmHg, DBP > 85 mmHg, or use of antihypertensive drugs	1.44 (0.94-2.21)	
	Hyperglycemia	FBS > 100 mg/dL or use of glucose-lowering drugs	1.48 (0.96-1.65)	
	Mets	IDF	1.87 (1.36-2.57)*	
	Elevated WC	WC 90 cm for men and 80 cm for women	9.27 (5.32-16.15)*	

NWO, Normal weight obesity, M, male, F, female, TC, total cholesterol, HOMA, Homeostatic Model Assessment for Insulin Resistance, LDL, low-density lipoproteins, HDL, high-density lipoproteins, TG, triglyceride, SBP, systolic blood pressure, DBP, diastolic blood pressure, BMI, body mass index, WC, waist circumference, Mets, metabolic syndrome, DM, diabetes mellitus, HTN, hypertension, CM, centimeters, IDF, International Diabetes Federation, OR, odds ratio, CI, Confidence Interval.

*Statistically significant (p-value < 0.05).

**OR calculated for NWO compare to NWNO.

^1^MetS is defined based on the criteria of IDF.

### Quantitative Synthesis

**Figure 2:**
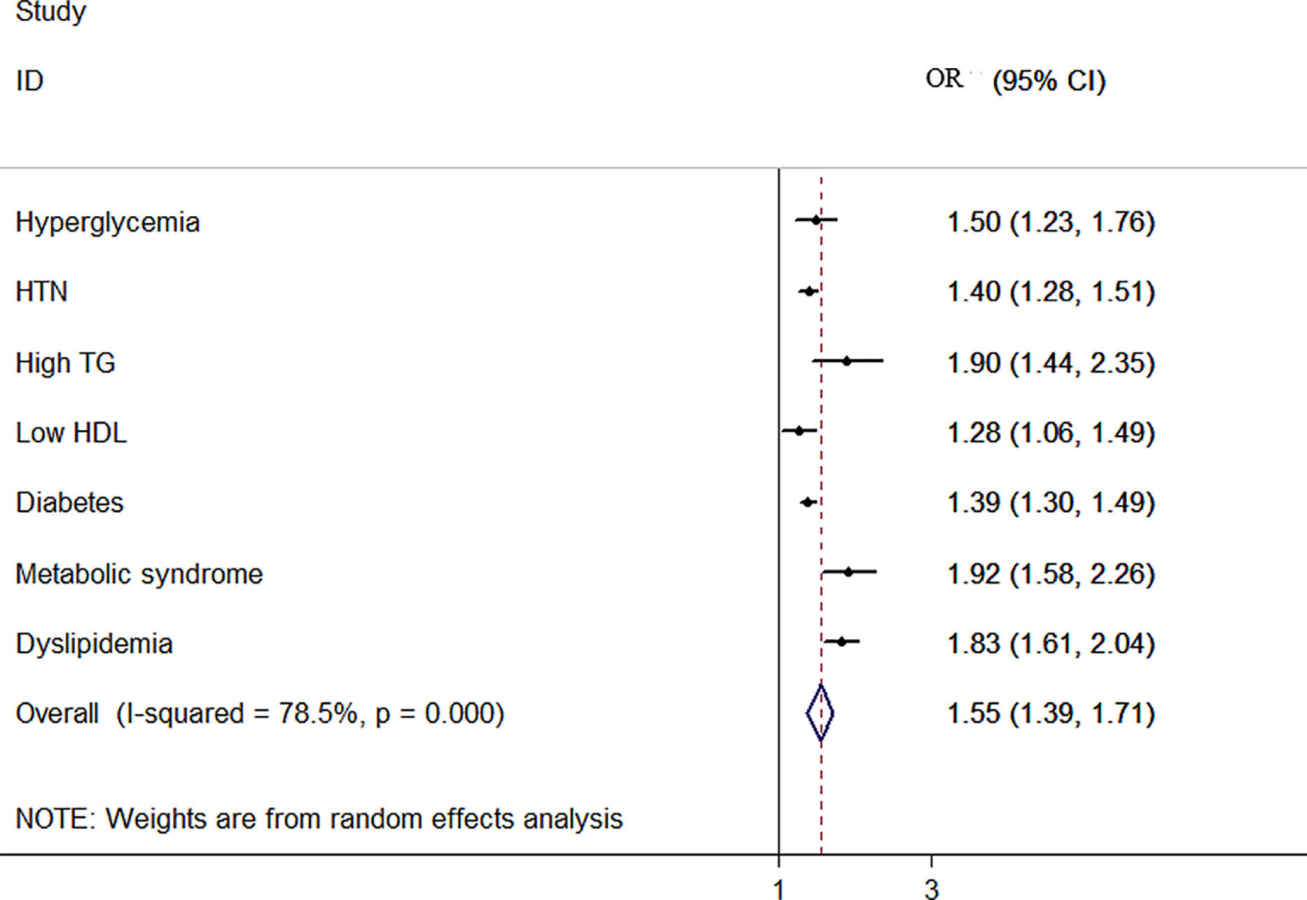
Forest plot detailing the pooled association between NWO with CMRFs.

The overall and sex-stratified pooled ORs of the relationship between NWO and CMRFs are shown in **Table 4**. The result of the meta-analysis showed that the overall odds ratio of hyperglycemia increased by 50% (OR:1.50, 95%:1.23, 1.76), of high TG by 90% (OR:1.90, 95% CH:1.44, 2.35), of low HDL by 28% (OR: 1.28, 95% CI:1.06, 1.49) and of diabetes by 39% (OR:1.39, 95% CI:1.30, 1.49) among NWO individuals. Also, the random effect meta-analysis showed increased odds of dyslipidemia by 83% (OR:1.83, 95% CI:1.61, 20.4), of HTN by 40% (OR:1.40, 95% CI:1.28, 1.51) and of metabolic syndrome by 92% (OR:1.92, 95% CI:1.58, 2.26) in the same population (**Figure 2)**.

**Table 4:** Stratified meta-analysis of association between NWO with CMRFs according to sex.

variables	No study	Sample size	Pooled odds ratio (95% CI)	Heterogeneity assessment		
				I-squared %	Model	P-value
Hyperglycemia						
Overall	6	6,934	1.50 (1.23, 1.76)*	0.00	Fixed	0.958
Both sexes	3	3,363	1.46 (1.15, 1.78)	0.00	Fixed	0.684
Female	3	3,571	1.58 (1.10, 2.07)*	0.00	Fixed	0.937
HTN						
Overall	13	155,397	1.40 (1.28, 1.51)*	57.30	Random	0.005
Both sexes	5	21,659	1.56 (1.35, 1.78)*	0.00	Fixed	0.587
Male	2	87,800	1.43 (0.96, 1.90)	89.50	Random	0.002
Female	6	45,938	1.25 (1.18, 1.33)*	25.80	Fixed	0.241
High TG						
Overall	6	6,934	1.90 (1.44, 2.35)*	0.00	Fixed	0.785
Both sexes	3	3,363	1.65 (1.05, 2.24)*	0.00	Fixed	0.746
Female	3	3,571	2.26 (1.55, 2.98)*	0.00	Fixed	0.924
Low HDL						
Overall	6	6,934	1.28 (1.06, 1.49)*	29.80	Fixed	0.212
Both sexes	3	3,363	1.38 (1.09, 1.67)*	38.20	Fixed	0.198
Female	3	3,571	1.15 (0.82, 1.47)	27.40	Fixed	0.252
Diabetes						
Overall	7	146,676	1.39 (1.30, 1.49)*	9.30	Fixed	0.358
Both sexes	2	16,438	1.49 (1.11, 1.87)*	45.5	Fixed	0.175
Male	2	87,800	1.35 (1.25, 1.45)*	0.00	Fixed	0.886
Female	3	42,438	1.62 (1.38, 1.86)*	0.00	Fixed	0.878
Metabolic syndrome						
Overall	6	36,854	1.92 (1.58, 2.26)*	68.40	Random	0.002
Both sexes	5	23,688	1.82 (1.38, 2.26)*	66.40	Random	0.018
Female	2	7,762	1.79 (1.44, 2.13)*	0.00	Fixed	0.878
Dyslipidemia						
Overall	7	135,276	1.83 (1.61, 2.04)*	80.00	Random	<0.001
Both sexes	3	2,737	1.73 (0.86, 2.60)*	45.70	Random	0.159
Male	2	87,800	2.23 (1.40, 3.06)*	92.80	Random	<0.001
Female	4	44,739	1.61 (1.52, 1.69)*	80.00	Random	<0.001

*Statistically significant (P-value < 0.05)

HDL, high-density lipoproteins; TG, triglyceride; HTN, hypertension; No, number; CI, confidence interval; CMRFs, cardio-metabolic risk factors; NWO, Normal weight obesity; HDL, high-density lipoproteins; TG, triglyceride; HTN, hypertension; No, number; CI, confidence interval; CMRFs, cardio-metabolic risk factors; NWO, Normal weight obesity.

**Figure 3:**
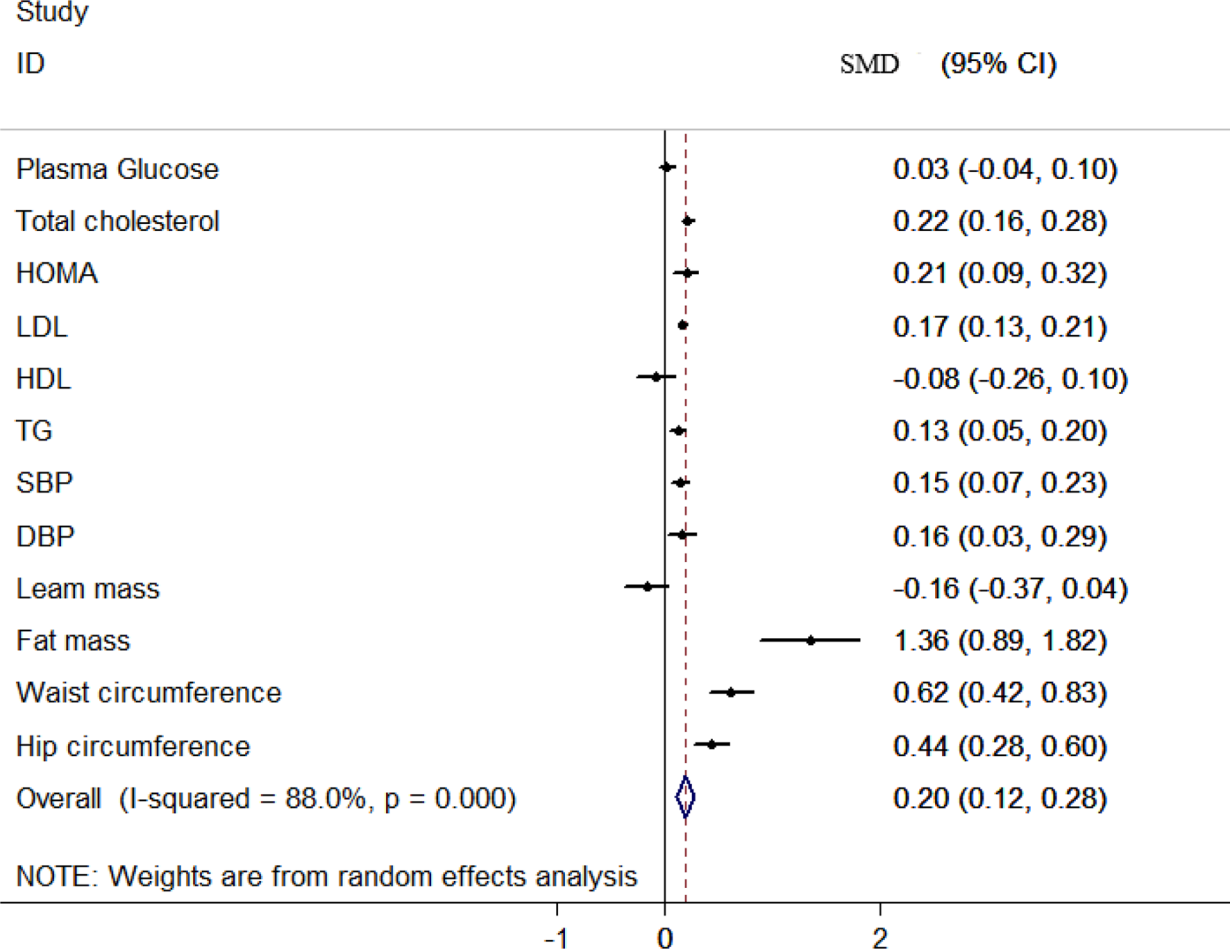
Forest plot detailing the pooled association between NWO with mean CMRFs.

The overall and sex-stratified association between NWO and the mean of CMRFs are shown in **Table 5**. A low to high heterogeneity was seen among included studies based on the CMRFs. Based on the fixed-effect model meta-analysis, the overall mean of TC (SMD: 0.22, 95% CI: 0.16, 0.28) and LDL (SMD: 17, 95% CI: 0.13, 0.12) was higher in NWO individuals compared to the normal weight none obese (NWNO) individuals. Based on the random effect meta-analysis, being NWO statistically increased the mean of HOMA (SMD: 0.12, 95% CI: 0.09, 0.32), TG (SMD: 0.13, 95% CI: 0.05, 0.20), SBP (SMD: 0.15, 95% CI: 0.07, 0.23), DBP (SMD: 0.16, 95% CI: 0.03, 0.29). However, the relationship between plasma glucose, HDL and lean mass was not statistically significant (**Figure 3**).

**Table 5:** Stratified meta-analysis of association between NWO with mean of CMRFs according to sex.

Variables	No study	Sample size	SMD (95% CI)	Heterogeneity assessment		
				I-squared %	Model	P-value
Plasma glucose						
Overall	9	12,312	0.03 (-0.04, 0.10)	66.30	Random	0.003
Both sexes	3	4,806	0.14 (0.07, 0.21)*	0.00	Fixed	0.410
Male	3	3,510	0.02 (-0.04, 0.09)	54.90	Fixed	0.109
Female	3	3,996	-0.05 (-0.11, 0.01)	0.00	Random	0.672
Total cholesterol						
Overall	5	6,277	0.22 (0.16, 0.28)*	0.00	Fixed	0.559
Both sexes	3	3,132	0.20 (0.13, 0.26)*	0.00	Fixed	0.976
HOMA						
Overall	5	10,306	0.21 (0.09, 0.32)*	73.10	Random	0.005
Both sexes	2	3,889	0.26 (0.14, 0.38)*	0.00	Fixed	0.765
Female	2	4,390	0.10 (-0.13, 0.34)	85.9	Random	0.008
LDL						
Overall	7	10,393	0.17 (0.13, 0.21)*	0.00	Fixed	0.503
Both	3	4,806	0.15 (0.09, 0.22)*	0.00	Fixed	0.437
Male	2	2,608	0.23 (0.15, 0.34)*	0.00	Fixed	0.465
Female	2	2,979	0.14 (0.06, 0.21)*	0.00	Fixed	0.865
HDL						
Overall	8	12,312	-0.08 (-0.26, 0.10)	94.70	Random	<0.001
Both sexes	3	4,806	-0.01 (-0.44, 0.41)	95.90	Random	<0.001
Male	2	3,510	-0.21 (-0.39, -0.03)	80.70	Random	0.023
Female	3	3,996	-0.08 (-0.20, 0.04)	94.70	Random	<0.001
TG						
Overall	9	12,312	0.13 (0.05, 0.20)*	70.30	Random	0.001
Both sexes	3	4,806	0.09 (-0.02, 0.21)	50.30	Fixed	0.133
Male	3	3,510	0.05 (-0.09, 0.12)	83.20	Random	0.003
Female	3	3,996	0.20 (0.13, 0.26)*	0.00	Fixed	0.858
SBP						
Overall	6	12,312	0.15 (0.07, 0.23)*	71.80	Random	<0.001
Both sexes	3	4,806	0.19 (-0.06, 0.44)	87.20	Random	<0.001
Male	3	3,510	0.14 (0.01, 0.28)*	70.80	Random	0.033
Female	3	3,996	0.12 (0.05, 0.19)*	55.20	Fixed	0.107
DBP						
Overall	5	10,393	0.16 (0.03, 0.29)*	86.50	Random	<0.001
Both sexes	3	4,806	0.10 (-0.18, 0.40)	90.60	Random	<0.001
Male	2	2,608	0.33 (0.004, 0.66)*	89.50	Random	0.002
Female	2	2,979	0.09 (0.01, 0.17)*	7.10	Fixed	0.300
Lean mass						
Overall	5	5,876	-0.16 (-0.37, 0.04)	90.20	Random	<0.001
Male	2	2,608	-0.22 (-0.62, 0.16)	92.60	Random	<0.001
Female	2	2,979	-0.06 (-0.14, 0.01)	0.00	Fixed	0.323
Fat mass						
Overall	6	7,795	1.36 (0.89, 1.82)*	98.50	Random	<0.001
Both sexes	2	2,208	0.79 (0.69, 0.88)*	12.00	Fixed	0.284
Male	2	2,608	1.75 (1.54, 1.95)*	66.30	Random	0.085
Female	2	2,979	1.63 (1.10, 2.17)*	95.50	Random	<0.001
Waist circumference						
Overall	8	12,246	0.62 (0.42, 0.83)*	95.80	Random	<0.001
Male	3	N.R	0.68 (0.61, 0.75)	0.716	Fixed	0.716
Female	4	N.R	0.71 (0.56, 86.50)	80.30	Random	0.002
Hip circumference						
Overall	5	12,246	0.44 (0.28, 0.60)*	91.00	Random	<0.001
Male	2	3,510	0.36 (0.24, 0.55)	57.80	Fixed	0.124
Female	2	6,297	0.49 (0.18, 0.80)	93.10	Random	<0.001

*Statistically significant (P-value < 0.05).

HOMA, Homeostatic Model Assessment for Insulin Resistance; LDL, low-density lipoproteins; HDL, high-density lipoproteins; TG, triglyceride; SBP, systolic blood pressure; DBP, diastolic blood pressure; HTN, hypertension; No, number; CI, confidence interval; CMRFs, cardio-metabolic risk factors; NWO, Normal weight obesity; HDL, high-density lipoproteins; TG, triglyceride; HTN, hypertension; No, number; CI, confidence interval; CMRFs, cardio-metabolic risk factors; NWO, Normal weight obesity; SMD, Standardized Mean Difference; N.R, Not Reported.

### Publication Bias

Except for HTN (coefficient =1.70, p-value=0.003), no publication bias was observed in articles studying the association between NWO and CMRFs for dichotomous and continuous data.

### Sensitivity Analysis

The sensitivity analysis result indicated that the pooled OR of the relationship between NWO and HTN was not substantially affected by each study (OR:1.23, 95% CI:1.19, 1.27).

## Discussion

To our knowledge, this is the first systematic review and meta-analysis that compared CMRFs among NWO and NWNO individuals across the entire population. We found 50% and 42% increased odds of hyperglycemia and diabetes among NWO individuals compared to the NWNOs, respectively. NWO individuals also have 40%, 83%, and 32% increased odds of HTN, dyslipidemia, and reduced HDL levels, respectively. Interestingly, NWO individuals also had an increased odds of hypertriglyceridemia as high as 90%. The results of other studies, such as Yu et al. (37) on CMRFs across various types of obesity, are comparable to that of ours. In the aforementioned study, the odds of hyperglycemia and diabetes were 40% and 103% in those with central obesity, 78% odds of HTN, and 142% hypertriglyceridemia (37). Although it seems that NWO imposes less a CMRF, some of its complications are comparable to that of obesity (central, general, and combined) (34); furthermore, despite being in a relatively better status than the obese, NWO individuals have a significantly greater CMRFs in comparison to the NWNOs. Moreover, the assessment of NWO-related comorbidities is of particular importance since the prevalence of NWO is exceptionally high (ranging from 5 to 45% based on sex, age, and the definition of NWO) (29, 38, 39). With a prevalence of 45% even among adolescents, NWO acts as a potent risk factor for future comorbidities; Hence, preventing and treating NWO can drastically reduce these comorbidities as well as obesity in adulthood; nonetheless, seemingly, due to lifestyle changes, unhealthy diets and lack of adequate physical activity, and sedentary lifestyle, the prevalence of NWO is increasing in a worrisome manner (40). However, these NWO individuals will go unnoticed and undiagnosed due to the inadequacies of BMI measurement, and despite their high body fat, due to their normal BMI levels, no treatment and preventive measure will be taken until it is too late. Furthermore, although obesity is a well-known associate of metabolic dysregulation, and there have been numerous studies on conditions that can result from obesity, yet the new concept of obesity (in which the weight itself is not as important as the body’s fat percent) is not well known nor studied. There have been studies on normal weight obesity regarding the conditions that can arise from it; however, compared to obesity, the number of studies are preliminary and more studies need to be done so that normal weight obesity gets the recognition that it needs as it is imperative for individuals to be well aware of their condition in order to take preventive measures. The public must be educated on the subject of NWO and must know that a normal BMI does not necessarily mean that they are not obese; in fact, they might have normal weight obesity, and regardless of their normal BMI, they are at increased risk of cardiometabolic conditions.

### Limitations and Strength

To the best of our knowledge, this is the first systematic review and meta-analysis comparing CMRFs among NWO and NWNO individuals in the entire population (age range 13 to 75), with a sample size of 177,792 proper research methods, it gives a realistic status of NWO globally. Our limitations were the use of manuscripts with an English full text. Furthermore, the unequal number of studies in different countries and differences in measurement and methodological aspects of the included studies resulting in high heterogeneity were among our limitations.

## Conclusion

The present study illustrated the significant odds of CMRFs among NWO individuals compared to subjects with NWNO. Indicating the inadequacy of the BMI measurement and the need for body fat assessment instead, for a better risk assessment. Furthermore, the necessity of preventive measures and interventions to significantly reduce the burden of the aforementioned condition is essential to avoid the upcoming obesity pandemic.

## Data Availability Statement

The raw data supporting the conclusions of this article will be made available by the authors, without undue reservation.

## Author Contributions

NK, MQ, and OT-M designed the study. NK and SN searched the databases. NK and SN screened and extracted the data. ES screened and analyzed the data. MQ, NK, OTM, RK, and MHB prepared the results. NK, MQ, and ZA wrote the paper. All other authors read and approved the final manuscript.

## Funding

This study was funded by Alborz University of Medical Sciences.

## Conflict of Interest

The authors declare that the research was conducted in the absence of any commercial or financial relationships that could be construed as a potential conflict of interest.

## Publisher’s Note

All claims expressed in this article are solely those of the authors and do not necessarily represent those of their affiliated organizations, or those of the publisher, the editors and the reviewers. Any product that may be evaluated in this article, or claim that may be made by its manufacturer, is not guaranteed or endorsed by the publisher.

## Abbreviations

NWO, Normal weight obesity; CMRFs, cardio-metabolic risk factors; NWNO, normal weight non obese; M, male; F, female; TC, total cholesterol; HOMA, Homeostatic Model Assessment for Insulin Resistance; LDL, low-density lipoproteins; HDL, high-density lipoproteins; TG, triglyceride; SBP, systolic blood pressure; DBP, diastolic blood pressure; BMI, body mass index; WC, waist circumference; MetS, metabolic syndrome; DM, diabetes mellitus; HTN, hypertension; CM, centimeters; IDF, International Diabetes Federation; SMD, Standardized Mean Difference; OR, odds ratio; SD, Standard Deviation; CI, Confidence Interval; Q.A, quality assessment.
