# Supplementary material for: Normal Weight Obesity and Cardiometabolic Risk Factors: A Systematic Review and Meta-Analysis
Source: Front Endocrinol (Lausanne). 2022 Mar 24;13:857930. doi: 10.3389/fendo.2022.857930 (PMC8987277; doi:10.3389/fendo.2022.857930)
Supplement: Supplementary file 1 [file Table_1.docx]

| **PubMed** |
| --- |
| (((("Normal Weight Obesity"[Title/Abstract]) OR "Normal BMI Obesity"[Title/Abstract]) OR "Central Obesity"[Title/Abstract]) OR "Obesity Phenotype"[Title/Abstract]) OR (((((("Regional Body Fat"[Title/Abstract]) OR "normal weight"[Title/Abstract]) OR "Normal Weight Phenotype"[Title/Abstract]) OR "Normal Body Mass Index" [Title/Abstract]) AND (("Obese"[Title/Abstract]) OR "Obesity"[Title/Abstract])) |
| **Scopus** |
| ( ( ( TITLE-ABS-KEY ( Normal Weight Obesity )  OR  TITLE-ABS-KEY ( *"* Normal BMI Obesity *"* )  OR  TITLE-ABS-KEY ( *"* Central Obesity *"* )  OR  TITLE-ABS-KEY ( *"* Obesity Phenotype *"* )  OR  TITLE-ABS-KEY ( *"* Regional Body Fat *"* )  OR  TITLE-ABS-KEY ( *NWO* ) ))) |
| **ISI/WOS** |
| TOPIC: (Normal Weight Obesity) OR TOPIC: ("Normal BMI Obesity ") OR TOPIC: ("Central Obesity ") OR TOPIC: ("diastolic pressure") OR TOPIC: ("Obesity Phenotype ") OR TOPIC: (Regional Body Fat) OR TOPIC: ("NWO")  Indexes=SCI-EXPANDED, SSCI, CPCI-S, CPCI-SSH, ESCI Timespan=All years |

**Supplementary Table 1: Search strategy**
